# Supplementary material for: Dopaminergic modulation of the exploration/exploitation trade-off in human decision-making
Source: eLife. 2020 Jun 2;9:e51260. doi: 10.7554/eLife.51260 (PMC7266623; doi:10.7554/eLife.51260)
Supplement: Transparent reporting form [file elife-51260-transrepform.docx]

***eLife’s* transparent reporting form**

We encourage authors to provide detailed information *within their submission* to facilitate the interpretation and replication of experiments. Authors can upload supporting documentation to indicate the use of appropriate reporting guidelines for health-related research (see [EQUATOR Network](http://www.equator-network.org/%20)), life science research (see the [BioSharing Information Resource](https://biosharing.org/)), or the [ARRIVE guidelines](http://www.plosbiology.org/article/info:doi/10.1371/journal.pbio.1000412) for reporting work involving animal research. Where applicable, authors should refer to any relevant reporting standards documents in this form.

If you have any questions, please consult our Journal Policies and/or contact us: [editorial@elifesciences.org](mailto:editorial@elifesciences.org).

**Sample-size estimation**

- You should state whether an appropriate sample size was computed when the study was being designed
- You should state the statistical method of sample size computation and any required assumptions
- If no explicit power analysis was used, you should describe how you decided what sample (replicate) size (number) to use

Please outline where this information can be found within the submission (e.g., sections or figure legends), or explain why this information doesn’t apply to your submission:

Information about sample-size estimation can be found in the ‘Materials and Methods’ section within the subsection ‘Participants’.

**Replicates**

- You should report how often each experiment was performed
- You should include a definition of biological versus technical replication
- The data obtained should be provided and sufficient information should be provided to indicate the number of independent biological and/or technical replicates
- If you encountered any outliers, you should describe how these were handled
- Criteria for exclusion/inclusion of data should be clearly stated
- High-throughput sequence data should be uploaded before submission, with a private link for reviewers provided (these are available from both GEO and ArrayExpress)

Please outline where this information can be found within the submission (e.g., sections or figure legends), or explain why this information doesn’t apply to your submission:

- number experiment repetitions: Methods section within the subsection ‘General procedure’.

- data availability: We will share all source code (modeling and statistical analysis) and all anonymized behavioral data and processed data (con-images) from fMRI analyses on OSF.io. See Data availability statement in SI. We will send you the corresponding link asap.

- outliers: not encountered

- criteria for exclusion/inclusion of data: no data excluded

**Statistical reporting**

- Statistical analysis methods should be described and justified
- Raw data should be presented in figures whenever informative to do so (typically when N per group is less than 10)
- For each experiment, you should identify the statistical tests used, exact values of N, definitions of center, methods of multiple test correction, and dispersion and precision measures (e.g., mean, median, SD, SEM, confidence intervals; and, for the major substantive results, a measure of effect size (e.g., Pearson's r, Cohen's d)
- Report exact p-values wherever possible alongside the summary statistics and 95% confidence intervals. These should be reported for all key questions and not only when the p-value is less than 0.05.

Please outline where this information can be found within the submission (e.g., sections or figure legends), or explain why this information doesn’t apply to your submission:

Analysis methods:

- computational model: Methods section, subsection ‘Computational modeling of explore/exploit behavior’, *Appendix 1* Methods section, subsections ‘Computational modeling (delta rule)’, ‘Fixed parameters (Bayesian learner)’

- model comparison: Methods section, subsection ‘Model comparison’

- fMRI-data analysis: Methods section, subsections ‘FMRI data analysis’, ‘First-level analysis’ and ‘Second-level analysis’

Results:

- behavioral performance: Results section, subsection ‘Participants learn to keep track of the best bandit’, and figure 2

- drug effects on model-free behavioral performance: Results section, subsection ‘No significant drug effects on model-free performance measures’

- model comparison: Figure 3

- drug effects on model-related measures of explore/exploit behavior: Results section, subsection ‘L-dopa reduces directed exploration’, figure 6 & Appendix – table 2 (mean of group-level posterior distributions), Appendix 1 – figure 1 & Appendix – table 2 (standard deviation of group-level posterior distributions), Appendix 1 – figure 2 (subject-level posterior distributions)

- brain activation associated with exploration/exploitation: Results section, subsection ‘Distinct brain networks orchestrate exploration and exploitation’, figure 7, Appendix 1 – figure 5, Appendix 1 – table 6, 7

- differential brain activation for random and directed exploration: Results section, subsection ‘Distinct brain networks orchestrate exploration and exploitation’, figure 7, Appendix 1 – figure 6

- coding of prediction errors: Appendix 1, Results section, subsection ‘Neural activation in response to model-based prediction errors (PEs)’, Appendix 1 – figure 7

- drug effects on explore/exploit brain activation (*classical analysis*): Results section, subsection ‘No evidence for a direct drug modulation of exploration/exploitation-related brain activation’

- drug effects on explore/exploit brain activation (*exploratory analysis*): Results section, subsection ‘L-dopa indirectly modulates exploration via reducing neural coding of overall uncertainty’, figure 8 & table 2

- test of inverted-U-shaped dopamine hypothesis: Appendix 1, Results section, Appendix 1 – figure 3, 4 & table 3, 4

(For large datasets, or papers with a very large number of statistical tests, you may upload a single table file with tests, Ns, etc., with reference to sections in the manuscript.)

**Group allocation**

- Indicate how samples were allocated into experimental groups (in the case of clinical studies, please specify allocation to treatment method); if randomization was used, please also state if restricted randomization was applied
- Indicate if masking was used during group allocation, data collection and/or data analysis

Please outline where this information can be found within the submission (e.g., sections or figure legends), or explain why this information doesn’t apply to your submission:

Session order (placebo, Ldopa, haloperidol) for each participant: Methods section, subsection ‘General procedure’

**Additional data files (“source data”)**

- We encourage you to upload relevant additional data files, such as numerical data that are represented as a graph in a figure, or as a summary table
- Where provided, these should be in the most useful format, and they can be uploaded as “Source data” files linked to a main figure or table
- Include model definition files including the full list of parameters used
- Include code used for data analysis (e.g., R, MatLab)
- Avoid stating that data files are “available upon request”

Please indicate the figures or tables for which source data files have been provided:

We will share all R code (modeling and statistical analysis) on OSF.io. Also we will make all (anonymized) behavioral as well as processed (con-images) fMRI data available on OSF.io. As soon as we have uploaded all data in a well-documented format, we will send you the corresponding link.
